# Complete Genome Sequence of Micrococcus luteus Strain CW.Ay, Isolated from Indoor Air in a Hong Kong School

**DOI:** 10.1128/mra.01194-21

**Published:** 2022-02-17

**Authors:** C. A. C. M. Wong, G. K. K. Lai, S. D. J. Griffin, F. C. C. Leung

**Affiliations:** a Shuyuan Molecular Biology Laboratory, The Independent Schools Foundation Academy, Hong Kong SAR, China; Montana State University

## Abstract

Micrococcus luteus strain CW.Ay was isolated from indoor air in Hong Kong. The complete genome (2,543,764 bp; GC content, 72.93%) was established by hybrid assembly and comprised a linear plasmid and a single chromosome featuring many genes to account for its broad distribution in very diverse habitats.

## ANNOUNCEMENT

Micrococcus luteus (formerly Micrococcus lysodeikticus) is a Gram-positive, nonmotile, obligate aerobe found in a remarkable range of habitats, including soil (1, 2), seawater (3, 4), freshwater (5, 6), and surfaces such as clothing and human skin (7, 8). It is also airborne in dust and bioaerosols and has been recovered from samples of indoor air (9, 10) and urban air (11). *Micrococcus* spp. even comprise a major proportion of bacteria recovered from the lower stratosphere (12). Given its mobility and the variety of conditions M. luteus may encounter, the features encoded by its small genome (∼2.5 Mbp) enable impressive versatility and resilience.

CW.Ay was isolated from indoor air in a school classroom in Cyberport, Hong Kong, using the IUL Spin Air Basic sampler passing 100 L/min onto the surface of Luria agar for 5 min. After incubation for 48 h at 27°C, selected yellow colonies were passaged 10 times on Luria agar. A single colony was incubated in Luria broth for 24 h before DNA extraction using a PureLink Genomic DNA Mini Kit (Invitrogen). Paired-end short-read sequencing libraries were prepared using the Nextera XT DNA library preparation kit and sequenced via the Illumina MiSeq platform using v3 chemistry (2 × 300 bp). Adapter sequences were removed using Trimmomatic v0.32 (13) and reads were quality filtered and trimmed, producing 703,669 read pairs, with an average length of 278 bp (∼196 Mbp). Long-read libraries, which were prepared from the same extracted DNA using the rapid barcoding kit SQK-RBK004, were sequenced using an Oxford Nanopore Technologies SpotON flow cell (vR9), MinION sequencer, and MinKNOW v3.1.8 software, with base calling by Guppy v2.1.3. The final long-read data set, trimmed by Porechop v0.2.4 (14, 15), totaled 182,541 reads (2.21 Gbp), with a mean length of 12,121 bp (*N*_50_, 20,668 bp). Default parameters were used for all software unless otherwise specified.

Assembly of short reads by Newbler v2.7 (Roche Diagnostics) suggested a draft genome of ∼2.5 Mbp, based on 411 contigs (mean length, 6,202 bp). However, Unicycler v0.4.3 (16) combined the Illumina and MinION data sets to render a circular chromosome of 2,449,847 bp and a linear plasmid of 93,917 bp (mean coverage, 194×), which were submitted to NCBI PGAP v5.0 (17) and PATRIC (18) for annotation.

Mash/MinHash using PATRIC (19) found the CW.Ay chromosome and plasmid to be close to Micrococcus luteus strain SA211 (GenBank accession number CP033200) and *Micrococcus* sp. strain A7 plasmid pLMA7 (GenBank accession number KJ599675.1), respectively, with average nucleotide identities of 97.11% and 97.09%, respectively (20).

Table 1 lists a selection of CW.Ay genes directed toward heavy metal resistance. In antimicrobial susceptibility tests (discs from Liofilchem), CW.Ay exhibited resistance to ampicillin (10 μg), chloramphenicol (30 μg), colistin (10 μg), erythromycin (15 μg), and sulfanilamide (30 μg), with relevant genes being chromosomally encoded (21).

**TABLE 1:** Heavy metal resistance genes in Micrococcus luteus CW.Ay

Gene(s)^*a*^	Protein(s)	Locus^*b*^	Reference(s)
*arsC*	Arsenate reductase (EC 1.20.4.4), thioredoxin coupled	10380	22
*arsC1-acr3-arsR-arsC-arsC*	Arsenic resistance operon, including arsenate-mycothiol transferase (EC 2.8.4.2) and arsenate efflux Acr3	05330–05350	22
*trxB-trxA*	Thioredoxin reductase (EC 1.8.1.9)-thioredoxin (similar to *arsT-arsX* in an arsenic-resistant *Microbacterium*)	11190–11195	22, 23
*arsO*	Flavin-dependent monooxygenase ArsO	08970	24
*czcD*	Cobalt/zinc/cadmium resistance protein	00780, 02990, 10120, 03020	25
*csoR-copZ*, *copZ*	Copper(I) chaperone CopZ	10385–10390, 06440	26
*copCD*	Periplasmic copper-binding proteins	09335–09350	27, 28
*merA*, *merR*, *merB*	Hg^2+^ reductase, organomercury lyase	10085–10100, 03005–03010	29
*cadD*	Cadmium resistance transporter	02990, 05315, 10110	30
*cadA*	Lead-, cadmium-, zinc-, and mercury-transporting ATPase (EC 7.2.2.21)	10150, 10175, 10395, 06435	31

a Gene assignments by NCBI PGAP v5.0 and UniProt.

b NCBI locus prefix K7G68_.

### Data availability.

The complete genome sequence and raw sequence data for Micrococcus luteus CW.Ay are available through NCBI under BioProject accession number PRJNA758605, with GenBank accession numbers CP082331 (chromosome) and CP082332 (plasmid) and SRA accession numbers SRX11980019 (MinION reads) and SRX11980018 (Illumina MiSeq rads).

## References

[1] Sims GK, Sommers LE, Konopka A. 1986. Degradation of pyridine by *Micrococcus luteus* isolated from soil. Appl Environ Microbiol 51:963–968. doi:10.1128/aem.51.5.963-968.1986. 16347070 PMC238995

[2] Ghosh A, Chaudhary SA, Apurva SR, Tiwari T, Gupta S, Singh AK, Katudia KH, Patel MP, Chikara SK. 2013. Whole-genome sequencing of *Micrococcus luteus* strain Modasa, of Indian origin. Genome Announc 1:e0007613. doi:10.1128/genomeA.00076-13. 23516205 PMC3593314

[3] Mohanrasu K, Premnath N, Siva Prakash G, Sudhakar M, Boobalan T, Arun A. 2018. Exploring multi potential uses of marine bacteria: an integrated approach for PHB production, PAHs and polyethylene biodegradation. J Photochem Photobiol B 185:55–65. doi:10.1016/j.jphotobiol.2018.05.014. 29864727

[4] Ferrari VB, Cesar A, Cayô R, Choueri RB, Okamoto DN, Freitas JG, Favero M, Gales AC, Niero CV, Saia FT, de Vasconcellos SP. 2019. Hexadecane biodegradation of high efficiency by bacterial isolates from Santos Basin sediments. Mar Pollut Bull 142:309–314. doi:10.1016/j.marpolbul.2019.03.050. 31232308

[5] López L, Pozo C, Rodelas B, Calvo C, Juárez B, Martínez-Toledo MV, González-López J. 2005. Identification of bacteria isolated from an oligotrophic lake with pesticide removal capacities. Ecotoxicology 14:299–312. doi:10.1007/s10646-003-6367-y. 15943106

[6] Min KR, Zimmer MN, Rickard AH. 2010. Physicochemical parameters influencing coaggregation between the freshwater bacteria *Sphingomonas natatoria* 2.1 and *Micrococcus luteus* 2.13. Biofouling 26:931–940. doi:10.1080/08927014.2010.531128. 21058055

[7] Chiereghin A, Felici S, Gibertoni D, Foschi C, Turello G, Piccirilli G, Gabrielli L, Clerici P, Landini MP, Lazzarotto T. 2020. Microbial contamination of medical staff clothing during patient care activities: performance of decontamination of domestic versus industrial laundering procedures. Curr Microbiol 77:1159–1166. doi:10.1007/s00284-020-01919-2. 32062686

[8] Khayyira AS, Rosdina AE, Irianti MI, Malik A. 2020. Simultaneous profiling and cultivation of the skin microbiome of healthy young adult skin for the development of therapeutic agents. Heliyon 6:e03700. doi:10.1016/j.heliyon.2020.e03700. 32337379 PMC7176942

[9] Kooken JM, Fox KF, Fox A. 2012. Characterization of *Micrococcus* strains isolated from indoor air. Mol Cell Probes 26:1–5. doi:10.1016/j.mcp.2011.09.003. 21963944 PMC4120257

[10] Kutmutia SK, Drautz-Moses DI, Uchida A, Purbojati RW, Wong A, Kushwaha KK, Putra A, Premkrishnan B, Heinle CE, Vettath VK, Junqueira A, Schuster SC. 2019. Complete genome sequence of *Micrococcus luteus* strain SGAir0127, isolated from indoor air samples from Singapore. Microbiol Resour Announc 8:e00646-19. doi:10.1128/MRA.00646-19. 31601656 PMC6787313

[11] Fang Z, Ouyang Z, Zheng H, Wang X, Hu L. 2007. Culturable airborne bacteria in outdoor environments in Beijing. Microb Ecol 54:487–496. doi:10.1007/s00248-007-9216-3. 17308950

[12] Smith DJ, Ravichandar JD, Jain S, Griffin DW, Yu H, Tan Q, Thissen J, Lusby T, Nicoll P, Shedler S, Martinez P, Osorio A, Lechniak J, Choi S, Sabino K, Iverson K, Chan L, Jaing C, McGrath J. 2018. Airborne bacteria in Earth's lower stratosphere resemble taxa detected in the troposphere: results from a new NASA aircraft bioaerosol collector (ABC). Front Microbiol 9:1752. doi:10.3389/fmicb.2018.01752. 30154759 PMC6102410

[13] Bolger AM, Lohse M, Usadel B. 2014. Trimmomatic: a flexible trimmer for Illumina sequence data. Bioinformatics 30:2114–2120. doi:10.1093/bioinformatics/btu170. 24695404 PMC4103590

[14] Wick RR. 2017. Porechop. https://github.com/rrwick/Porechop.

[15] Wick RR, Judd LM, Holt KE. 2018. Deepbinner: demultiplexing barcoded Oxford Nanopore reads with deep convolutional neural networks. PLoS Comput Biol 14:e1006583. doi:10.1371/journal.pcbi.1006583. 30458005 PMC6245502

[16] Wick RR, Judd LM, Gorrie CL, Holt KE. 2017. Unicycler: resolving bacterial genome assemblies from short and long sequencing reads. PLoS Comput Biol 13:e1005595. doi:10.1371/journal.pcbi.1005595. 28594827 PMC5481147

[17] Haft DH, DiCuccio M, Badretdin A, Brover V, Chetvernin V, O'Neill K, Li W, Chitsaz F, Derbyshire MK, Gonzales NR, Gwadz M, Lu F, Marchler GH, Song JS, Thanki N, Yamashita RA, Zheng C, Thibaud-Nissen F, Geer LY, Marchler-Bauer A, Pruitt KD. 2018. RefSeq: an update on prokaryotic genome annotation and curation. Nucleic Acids Res 46:D851–D860. doi:10.1093/nar/gkx1068. 29112715 PMC5753331

[18] Brettin T, Davis JJ, Disz T, Edwards RA, Gerdes S, Olsen GJ, Olson R, Overbeek R, Parrello B, Pusch GD, Shukla M, Thomason JA, III, Stevens R, Vonstein V, Wattam AR, Xia F. 2015. RASTtk: a modular and extensible implementation of the RAST algorithm for building custom annotation pipelines and annotating batches of genomes. Sci Rep 5:8365. doi:10.1038/srep08365. 25666585 PMC4322359

[19] Ondov BD, Treangen TJ, Melsted P, Mallonee AB, Bergman NH, Koren S, Phillippy AM. 2016. Mash: fast genome and metagenome distance estimation using MinHash. Genome Biol 17:132. doi:10.1186/s13059-016-0997-x. 27323842 PMC4915045

[20] Yoon SH, Ha SM, Lim J, Kwon S, Chun J. 2017. A large-scale evaluation of algorithms to calculate average nucleotide identity. Antonie Van Leeuwenhoek 110:1281–1286. doi:10.1007/s10482-017-0844-4. 28204908

[21] Alcock BP, Raphenya AR, Lau TTY, Tsang KK, Bouchard M, Edalatmand A, Huynh W, Nguyen A-LV, Cheng AA, Liu S, Min SY, Miroshnichenko A, Tran H-K, Werfalli RE, Nasir JA, Oloni M, Speicher DJ, Florescu A, Singh B, Faltyn M, Hernandez-Koutoucheva A, Sharma AN, Bordeleau E, Pawlowski AC, Zubyk HL, Dooley D, Griffiths E, Maguire F, Winsor GL, Beiko RG, Brinkman FSL, Hsiao WWL, Domselaar GV, McArthur AG. 2020. CARD 2020: antibiotic resistome surveillance with the Comprehensive Antibiotic Resistance Database. Nucleic Acids Res 48:D517–D525. doi:10.1093/nar/gkz935. 31665441 PMC7145624

[22] Sher S, Hussain SZ, Rehman A. 2020. Phenotypic and genomic analysis of multiple heavy metal-resistant *Micrococcus luteus* strain AS2 isolated from industrial waste water and its potential use in arsenic bioremediation. Appl Microbiol Biotechnol 104:2243–2254. doi:10.1007/s00253-020-10351-2. 31927763

[23] Achour-Rokbani A, Cordi A, Poupin P, Bauda P, Billard P. 2010. Characterization of the *ars* gene cluster from extremely arsenic-resistant *Microbacterium* sp. strain A33. Appl Environ Microbiol 76:948–955. doi:10.1128/AEM.01738-09. 19966021 PMC2813031

[24] Wang L, Chen S, Xiao X, Huang X, You D, Zhou X, Deng Z. 2006. *arsRBOCT* arsenic resistance system encoded by linear plasmid pHZ227 in *Streptomyces* sp. strain FR-008. Appl Environ Microbiol 72:3738–3742. doi:10.1128/AEM.72.5.3738-3742.2006. 16672525 PMC1472359

[25] Fierros-Romero G, Gómez-Ramírez M, Sharma A, Pless RC, Rojas-Avelizapa NG. 2020. *czcD* gene from *Bacillus megaterium* and *Microbacterium liquefaciens* as a potential nickel-vanadium soil pollution biomarker. J Basic Microbiol 60:22–26. doi:10.1002/jobm.201900323. 31692013

[26] Chaplin AK, Tan BG, Vijgenboom E, Worrall JA. 2015. Copper trafficking in the CsoR regulon of *Streptomyces lividans*. Metallomics 7:145–155. doi:10.1039/c4mt00250d. 25409712

[27] Lawton TJ, Kenney GE, Hurley JD, Rosenzweig AC. 2016. The CopC family: structural and bioinformatic insights into a diverse group of periplasmic copper binding proteins. Biochemistry 55:2278–2290. doi:10.1021/acs.biochem.6b00175. 27010565 PMC5260838

[28] Abriata LA, Banci L, Bertini I, Ciofi-Baffoni S, Gkazonis P, Spyroulias GA, Vila AJ, Wang S. 2008. Mechanism of Cu(A) assembly. Nat Chem Biol 4:599–601. doi:10.1038/nchembio.110. 18758441 PMC2596924

[29] Christakis CA, Barkay T, Boyd ES. 2021. Expanded diversity and phylogeny of *mer* genes broadens mercury resistance paradigms and reveals an origin for MerA among thermophilic archaea. Front Microbiol 12:682605. doi:10.3389/fmicb.2021.682605. 34248899 PMC8261052

[30] Crupper SS, Worrell V, Stewart GC, Iandolo JJ. 1999. Cloning and expression of *cadD*, a new cadmium resistance gene of *Staphylococcus aureus*. J Bacteriol 181:4071–4075. doi:10.1128/JB.181.13.4071-4075.1999. 10383976 PMC93898

[31] Nucifora G, Chu L, Misra TK, Silver S. 1989. Cadmium resistance from *Staphylococcus aureus* plasmid pI258 *cadA* gene results from a cadmium-efflux ATPase. Proc Natl Acad Sci USA 86:3544–3548. doi:10.1073/pnas.86.10.3544. 2524829 PMC287174

